# Endovascular treatment for basilar artery occlusion: a cost-effectiveness analysis based on a meta-analysis

**DOI:** 10.3389/fneur.2023.1267554

**Published:** 2023-10-20

**Authors:** Li Wang, Ying Yu, Limei Zhou, Ping Xu, Xianbin Guo, Yu Xie, Junxiu Cai, Min Pan, Jie Tang, Qingtao Gong, Rong Su, Yake Lou, Yan Liu

**Affiliations:** ^1^Department of Neurology, Zigong Third People’s Hospital, Zigong, China; ^2^Department of Interventional Neuroradiology, Beijing Tiantan Hospital, Capital Medical University, Beijing, China; ^3^Department of Cardiology, The Second Affiliated Hospital of Chongqing Medical University, Chongqing, China

**Keywords:** cost effectiveness analysis, endovascular treatment, basilar artery occlusion, stroke, meta-analysis

## Abstract

**Objective:**

This study aimed to investigate the efficacy and economic effect of endovascular treatment (EVT) combined with standard medical treatment (SMT) vs. SMT alone in Chinese patients with basilar artery occlusion (BAO) from the perspective of the Chinese healthcare system.

**Methods:**

We conducted a cost-effectiveness analysis using the results from a meta-analysis comparing EVT and SMT efficacy in Chinese patients with BAO-induced stroke using direct medical costs from the China National Stroke Registry. The meta-analysis’s primary outcome was excellent functional outcome (mRS scores of 0–2), with secondary outcomes being poor functional outcome (mRS scores of 3–5) and death (mRS score of 6). To compare EVT plus SMT’s cost-effectiveness with that of SMT alone, we constructed a combined decision tree and Markov model with a lifetime duration and a 3-month cycle length. The primary cost-effectiveness outcome was the incremental cost-effectiveness ratio (ICER), representing the incremental cost per incremental quality-adjusted life year (QALY). EVT was considered cost-effective if the ICER was lower than the willingness-to-pay (WTP) threshold of three times the *per capita* gross domestic product (GDP) in 2021 in China; otherwise, it would not be cost-effective.

**Results:**

The meta-analysis results indicated that EVT could increase the incidence of excellent functional outcomes, with a risk ratio (RR) of 2.23 (95% confidence interval, CI, 1.18–4.21), *p* = 0.01. Simultaneously, EVT reduced the risk of poor functional outcome and mortality in the EVT group, with RRs of 0.83 (95% CI, 0.67–1.03), *p* = 0.09, and 0.71 (95% CI, 0.59–0.85), *p* = 0.0002, respectively. The study also found that EVT plus SMT resulted in a lifetime effectiveness of 2.15 QALY (3.88 life years) for 32,213 international dollars (Intl.$) per patient with BAO. In contrast, SMT alone achieved an effectiveness of 1.46 QALY (3.03 life years) with a total cost of Intl.$ 13,592 per patient. The ICER was Intl.$ 27,265 per QALY (Intl.$ 22,098 per life-year), which fell below the WTP threshold.

**Conclusion:**

Compared to SMT, EVT improves the prognosis of BAO-induced stroke. Considering the Chinese healthcare system, adding EVT to SMT proves to be cost-effective for patients with BAO compared to SMT alone.

## 1. Introduction

Stroke significantly contributes to death and neurological disability among Chinese residents, imposing a substantial financial burden on society and families (1, 2). This burden has been exacerbated in recent years owing to population aging and insufficient awareness of vascular risk factor management, including tobacco abuse, hypertension, diabetes mellitus, and dyslipidemia, particularly in low- and middle-income countries (1, 3). In 2019, China recorded 3.94 million new stroke cases and 2.19 million stroke-related deaths, according to the Global Burden of Disease Study 2019 (2). Ischemic stroke accounts for over 80% of all strokes, and effective clinical management during the acute phase is critical for its prognosis (2, 4). Timely reperfusion therapy, aimed at preserving the ischemic penumbra, is the key to improving outcomes in acute ischemic stroke (AIS) caused by large vessel occlusion (LVO) (5).

The European Stroke Organization (ESO) guidelines recommend endovascular treatment (EVT) for patients with AIS, owing to LVO in the anterior circulation within 24 h of onset, as EVT offers significant benefits regarding functional improvement and reduced mortality when compared to standard medical therapy (SMT) (6). However, individuals with AIS resulting from basilar artery occlusion (BAO) typically experience severe clinical symptoms and swift deterioration, including quadriplegia and diminished consciousness. This decline results from acute ischemia affecting critical brain regions, specifically the cerebellum, brainstem, and thalamus, further exacerbated by inadequate collateral compensation from basilar artery perforating branches (7). Previous randomized controlled trials (RCTs) investigating EVT for acute BAO have failed to support the unequivocal superiority of EVT over SMT (8, 9), possibly because the best patients for EVT have not been identified. Subgroup analysis of the BASICS trial demonstrated that EVT was superior to SMT in patients with BAO and NIHSS scores >10 (9). Recently, the results of the two RCTs revealed that EVT had better functional outcomes than SMT at 90 days in selected patients with BAO, potentially marking a new chapter in the treatment of AIS attributable to BAO (10, 11).

However, the cost-effectiveness of performing EVT on patients with acute BAO within the Chinese healthcare system remains uncertain, largely because of the high costs of EVT in developing countries such as China. Furthermore, studies from various countries have reported varying levels of EVT effectiveness in treating BAO (8–11). Therefore, we conducted a cost-effectiveness analysis based on meta-analysis results to investigate EVT’s efficacy in patients with BAO and to assess its economic effect on the Chinese healthcare system.

## 2. Methods

### 2.1. Meta-analysis

#### 2.1.1. Search strategy

To identify potentially eligible citations, we searched PubMed, Embase, and Cochrane databases using keywords such as endovascular treatment, basilar artery occlusion, posterior circulation, stroke, mechanical thrombectomy, stent retriever, and embolectomy. We employed a search filter for RCT, which was obtained from the Harvard Council Library. This filter has a sensitivity of over 99% and is freely accessible at https://guides.library.harvard.edu/c.php?g=309982&p=2079544. In addition, we restricted our search to citations published in English.

#### 2.1.2. Inclusion and exclusion criteria

The following were the inclusion criteria: (1) randomized controlled trials, (2) patients with BAO, (3) both efficacy and safety outcomes at 90 days were reported, (4) a sample size of >50 patients, (5) the intervention was EVT, and the control was SMT, and (6) age not less than 18 years. Animal studies, retrospective studies, and cohort studies were excluded.

#### 2.1.3. Data extraction

Two authors (Wang and Yu) independently screened the eligible studies and extracted their baseline characteristics and outcome data. The quality of the studies was independently assessed by two other authors using the Cochrane Handbook for Systematic Reviews of Interventions (version 5.1.0). Disagreements were resolved by the fifth author (Lou). In cases of incomplete data, the corresponding author was contacted via email to obtain any missing information.

#### 2.1.4. Outcomes

The primary outcome of this study was an excellent functional outcome (defined as an mRS score of 0–2 at 90 days), and secondary outcomes included poor functional outcome (defined as an mRS score of 3–5 at 90 days) and mortality (mRS score 6).

#### 2.1.5. Statistical analysis

Statistical analyses were conducted using the Review Manager software (RevMan; The Cochrane Collaboration, Copenhagen, Denmark) version 5.3. We used the risk ratio (RR) and 95% confidence interval (CI) to compare the efficacies of EVT and SMT. A fixed-effects model was applied when the heterogeneity across studies was <50%, and a random-effects model was employed when the heterogeneity was ≥50%.

### 2.2. Cost-effectiveness analysis

#### 2.2.1. Model overview

**Figure 1:**
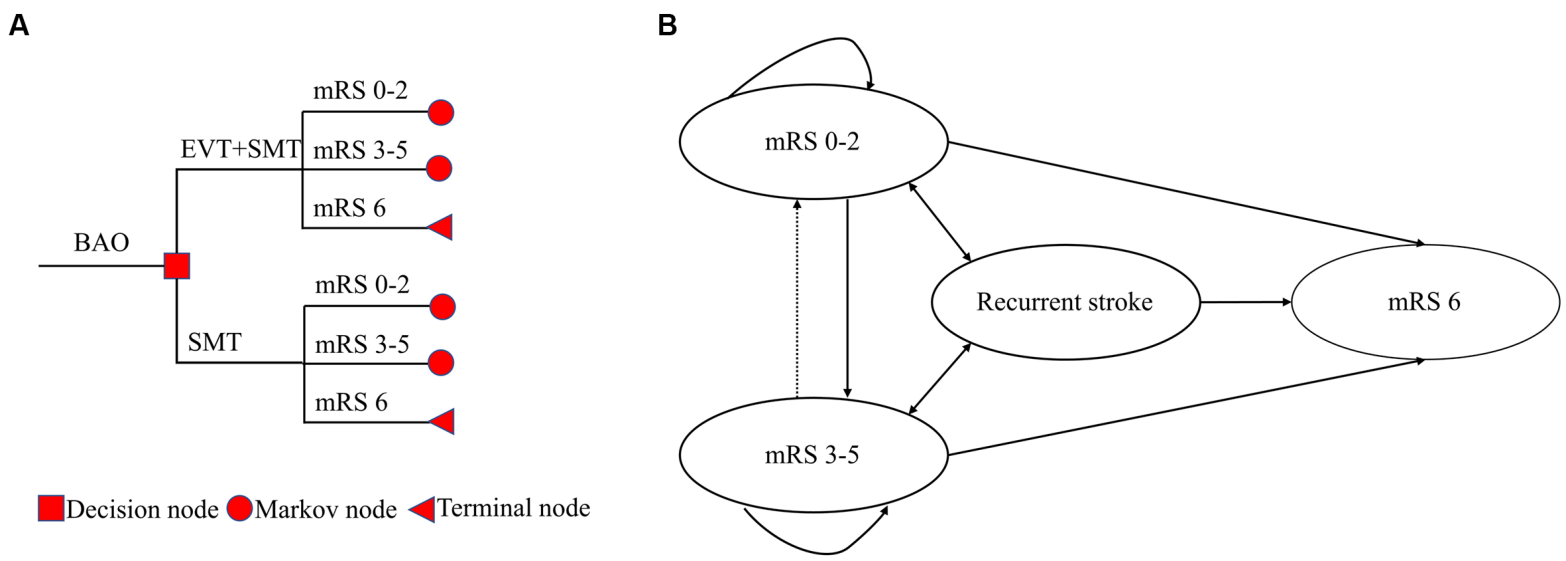
A combined short-term decision tree **(A)** and long-term Markov model **(B)**. The decision tree spanned 3 months, and the Markov model had 79 cycles, with each cycle lasting 3 months. The total simulation period was 20 years.

A combined short-term decision tree and long-term Markov model were constructed to simulate cost-effectiveness from the perspective of the Chinese healthcare system. In the decision tree, patients with BAO were randomized to receive either SMT + EVT or SMT alone. Patients in the decision tree entered one of the three states: “mRS 0–2,” “mRS 3–5,” or “mRS 6,” and lasted until 3 months. The proportion of patients in each state varied between the two groups because of different treatment strategies. Subsequently, patients from these states entered a Markov model and cycled until either 20 years elapsed or death occurred. The cycle length was set at 3 months, and the simulation period spanned the patient’s lifetime. However, since the mean age of the participants in the ATTENTION study was 66 years, simulating a 20-year period exceeds China’s average life expectancy, currently approximately 78 years. The Markov model had three transition states (“mRS 0–2,” “mRS 3–5,” and “recurrent stroke”) and one absorbed state (“Dead”). Patients in the Markov model could transition between states, with the cycle terminating upon entering the “Dead” state. The decision tree and Markov model were validated in other studies; details are shown in Figure 1 (12–14).

This study was reported according to the CHEERS 2022 statement (15).

#### 2.2.2. Transition probabilities

The proportions of “mRS 0–2,” “mRS 3–5,” and “mRS 6” at month 3 were derived from the meta-analysis (Supplementary Table 1). For transition probabilities in the Markov model, some were drawn from a community cohort investigating the clinical outcomes of post-stroke patients, while others were from the China National Stroke Registry (CNSR) (12, 16–19). Both groups were assumed to share similar transition probabilities after discharge. The yearly rate was converted to a 3-month transition probability using the formula “3-month transition probability = 1-exp (−yearly rate/4)” (12). The details of the transition probabilities are presented in Supplementary Table 2.

Considering that non-stroke mortality was higher in patients with disabling stroke than in those with non-disabling stroke, the RR was employed to adjust for this increased risk (20). Patients with non-disabling stroke were assumed to have a similar risk as the general population of the same age (20). The non-stroke mortality rate of the general population was obtained from the China Health Statistics Yearbook 2022 (21).

#### 2.2.3. Cost

Direct medical costs were estimated using a micro-costing approach. Costs not denominated in 2021 Chinese Yuan (CNY) were converted using China’s healthcare consumer price index from 2015 to 2021 (1.027, 1.038, 1.06, 1.043, 1.024, 1.018, and 1.004, respectively). To account for different purchasing powers in various countries, CNY was converted to international dollars (Intl.$) using purchasing power parity exchange rates of 4.19, providing a more accurate reflection of the purchasing power cost (22). All costs beyond 1 year were discounted at a rate of 0.05, ranging from 0 to 0.08, according to the China Guidelines for Pharmacoeconomic Evaluations (23).

The cost of EVT was obtained from a survey in China, representing the overall EVT cost in the country (24). It was observed that SMT cost varied with the mRS grades of patients: Intl.$ 2,718 for those with mRS 0–2 and Intl.$ 3,375 for those with mRS 3–5 (20). The costs of recurrent stroke events and annual post-hospitalization costs were assessed using data from the CNSR (24) (Supplementary Table 1).

#### 2.2.4. Utility

Utility scores were assigned to each disability state using the European Quality of Life Scale and the Chinese preference weights (17, 25). These scores were employed in calculating quality-adjusted life years (QALYs) by multiplying the total number of life years (LY) spent in a particular health state by the corresponding utility score. The utilities for mRS 0–2, mRS 3–5, and mRS 6 were 0.76 (95% CI, 0.69–0.82), 0.21 (95% CI, 0.17–0.26), and 0, respectively. A utility score of 0.20 (95% CI, 0.16–0.26) was applied for recurrent stroke. All utility scores were discounted at a rate of 0.05, ranging from 0 to 0.08, according to the China Guidelines for Pharmacoeconomic Evaluations.

#### 2.2.5. Primary outcomes

The primary outcome was the incremental cost-effectiveness ratio (ICER) of EVT plus SMT vs. SMT alone, representing the incremental cost (Intl.$) per incremental QALY. EVT was considered highly cost-effective if the ICER was lower than the willingness-to-pay (WTP) threshold of Intl.$ 19,326/QALY, which was once *per capita* gross domestic product (GDP) in 2021 China. It was considered cost-effective if the ICER ranged between Intl.$ 19,326–57,978/QALY and not cost-effective if the ICER exceeded the WTP threshold of Intl.$ 57,978/QALY, according to the China Guidelines for Pharmacoeconomic Evaluations. Secondary outcomes included total cost, incremental cost, total effectiveness, incremental effectiveness, ICER across different time horizons, and ICER without considering quality of life.

#### 2.2.6. Scenario analysis and sensitivity analysis

We performed a scenario analysis using different data sources, including the proportions of mRS scores of 0–2, 3–5, and 6 at 90 days in all patients with BAO (8–11), and the effectiveness of each RCT and EVT cost plugged into the model at 150 and 200% of the current cost.

To test the robustness of the results, we performed a one-way sensitivity analysis involving fluctuating input parameters within a given interval. A tornado diagram is used to display the results. For probabilistic sensitivity analysis (PSA), we performed 10,000 Monte Carlo simulations using probabilistic sensitivity sampling to test the uncertainty of the results. All costs were modeled using a γ distribution, utilities using a β distribution, and transition probabilities using a Dirichlet distribution. The PSA results were visualized through a scatter plot and acceptability curve.

#### 2.2.7. Standard protocol approvals, registrations, and patient consents

Our meta-analysis was registered in the PROSPERO database under registration number CRD42022357718. This study did not involve human participants; therefore, institutional review board approval or consent was not required.

## 3. Results

### 3.1. Meta-analysis

The meta-analysis results indicated that EVT increased the incidence of excellent functional outcomes, with an RR of 2.23 (95% CI, 1.18–4.21, *p* = 0.01). Simultaneously, the EVT group showed a reduced risk of poor functional outcomes and mortality, with RRs of 0.83 (95% CI, 0.67–1.03, *p* = 0.09) and 0.71 (95% CI, 0.59–0.85, *p* = 0.0002), respectively (Table 1).

**Table 1:** Results of the meta-analysis of EVT in Chinese patients with BAO.

mRS classifications	RR (95% CI)	*p*-value
mRS 0–2	2.23 (1.18–4.21)	0.01
mRS 3–5	0.83 (0.67, 1.03)	0.09
mRS 6	0.71 (0.59, 0.85)	0.0002

BAO, basilar artery occlusion; mRS, modified Rankin Scale; RR, risk ratio; CI, confidence interval.

### 3.2. Cost-effectiveness analysis

#### 3.2.1. Base case analysis

Over a lifetime simulation, each patient with BAO who received EVT plus SMT would gain 2.15 QALYs (3.88 LYs) at the cost of Intl.$ 32,213. Conversely, if only SMT was administered, the effectiveness would be 1.46 QALYs (3.03 LYs) with a total cost of Intl.$ 13,592, resulting in an ICER of Intl.$ 27,265/QALY (Intl.$ 22,098/LY) (Table 2). Notably, this value is one to three times China’s *per capita* GDP in 2021.

**Table 2:** Results of base case and scenario analysis.

Scenario	Total cost (Intl.$)	Incre-cost (Intl.$)	Total eff (QALY)	Incre eff (QALY)	ICER (Intl.$/QALY)	Total efficacy (LY)	Increased efficacy (LY)	ICER (Intl.$/LY)
*Base case analysis*								
SMT	13,592	–	1.46	–	–	3.03	–	–
EVT + SMT	32,213	18,621	2.15	0.68	27,265	3.88	0.84	22,098
*Scenario 1: Effectiveness of EVT from all BAO patients*								
SMT	13,683	–	1.57	–	–	3.12	–	–
EVT + SMT	32,025	18,343	2.13	0.56	32,877	3.83	0.70	26,020
*Scenario 2: Effectiveness of EVT from ATTENTION study*								
SMT	12,063	–	1.12	–	–	2.49	–	–
EVT + SMT	32,248	20,184	2.12	1.00	20,138	3.87	1.38	14,576
*Scenario 3: Effectiveness of EVT from BAOCHE study*								
SMT	14,101	–	1.43	–	–	3.13	–	–
EVT + SMT	32,816	18,715	2.32	0.89	21,131	4.12	0.99	18,972
*Scenario 4: Effectiveness of EVT from BEST study using ITT analysis*								
SMT	14,885	–	1.89	–	–	3.58	–	–
EVT + SMT	32,456	17,571	2.13	0.24	72,918	3.92	0.34	51,515
*Scenario 5: Effectiveness of EVT from BEST study using as-treated analysis*								
SMT	13,929	–	1.56	–	–	3.16	–	–
EVT + SMT	33,004	19,075	2.33	0.78	24,514	4.17	1.00	19,051
*Scenario 6: 150% of current EVT cost*								
SMT	13,592	–	1.46	–	–	3.03	–	–
EVT + SMT	40,560	26,968	2.15	0.68	39,487	3.88	0.84	32,004
*Scenario 7: 200% of current EVT cost*								
SMT	13,592	–	1.46	–	–	3.03	–	–
EVT + SMT	48,907	35,315	2.15	0.68	51,709	3.88	0.84	41,909

Intl.$, international dollar; Incre, incremental; Eff, effectiveness; QALY, quality-adjusted life year; LY, life year; SMT, standard medical therapy; EVT, endovascular treatment; ICER, incremental cost-effectiveness ratio; RCT, randomized controlled trials; BAOCHE study, Basilar Artery Occlusion Chinese Endovascular Trial; BEST study, Acute Basilar Artery Occlusion: Endovascular Interventions vs. Standard Medical Treatment; ATTENTION study, Endovascular Treatment for Acute Basilar Artery Occlusion; BASIC study, Basilar Artery International Cooperation Study; ITT, intention-to-treat.

**Figure 2:**
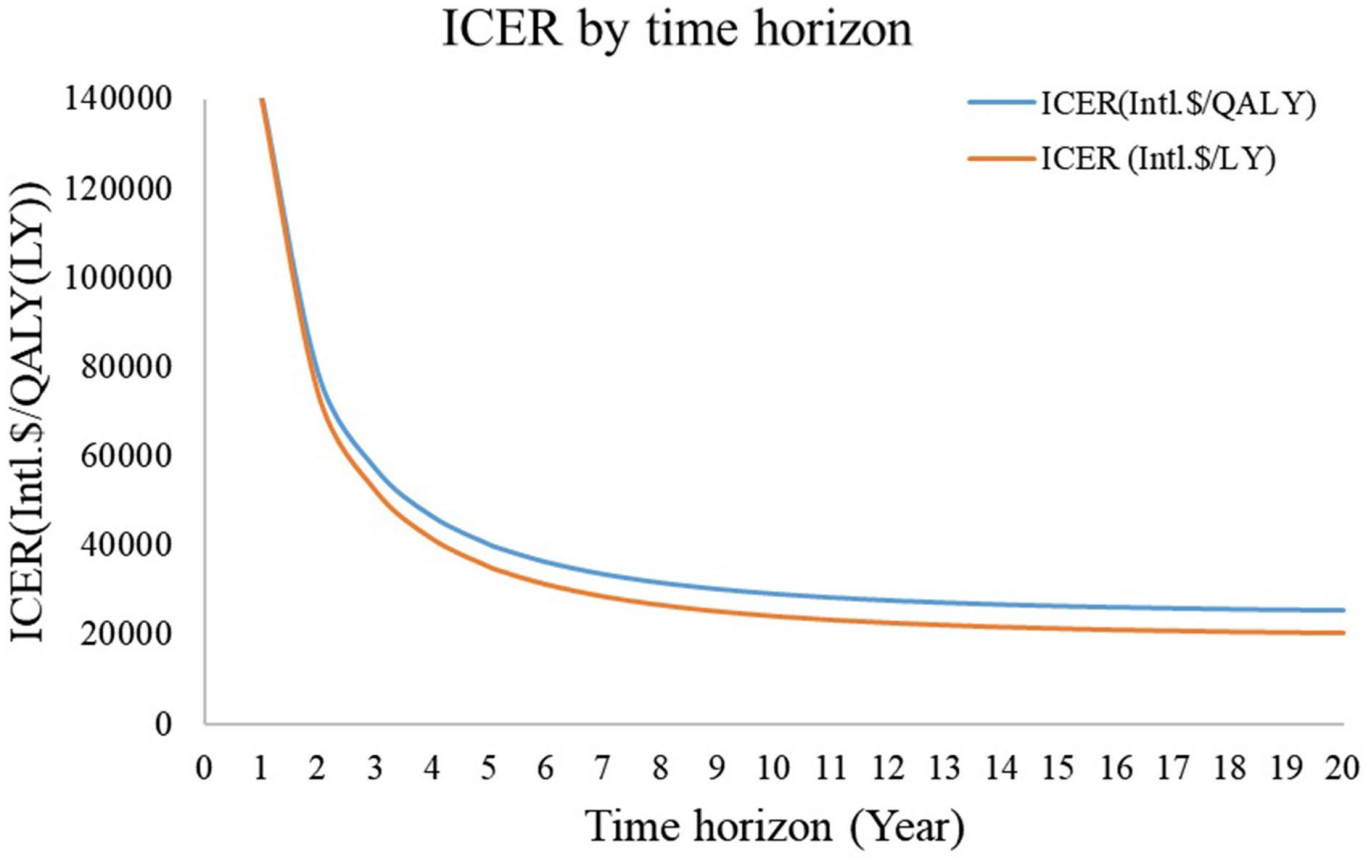
ICER by time horizon. When the time horizon was less than 3 years, the ICER of endovascular treatment plus standard medical therapy vs. standard medical therapy was higher than three times the *per capita* gross domestic product. ICER: incremental cost-effectiveness ratio.

The ICER across different time horizons revealed that, when life expectancy was no less than 3 years, the ICER of EVT plus SMT vs. SMT alone was lower than that of Intl.$ 57,978/QALY (Figure 2).

#### 3.2.2. Scenario analysis and sensitivity analysis

As shown in Table 2, scenario analysis based on different EVT costs and EVT plus SMT’s effectiveness vs. SMT consistently indicated that the ICER of EVT plus SMT vs. SMT alone was less than three times the *per capita* GDP in 2021 in China. The only exception was when the results of the BEST study with intention-to-treat analysis were employed in the Markov model.

**Figure 3:**
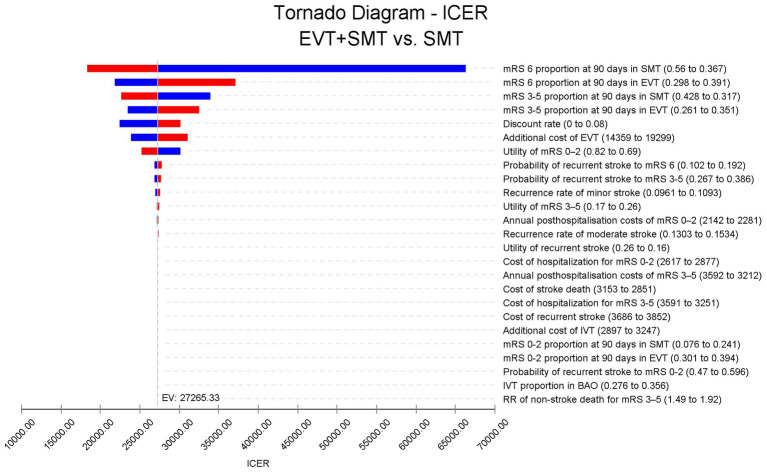
Tornado diagram. The mortality rate at 90 days in the standard medical therapy group and the endovascular treatment plus standard medical therapy group had the largest effect on the ICER fluctuation. Other parameters had a minimal effect on the ICER. ICER: incremental cost-effectiveness ratio.

A one-way sensitivity analysis revealed that the mRS proportion at 90 days in the SMT group had the most significant effect on the ICER. If the 90-day mortality rate in the SMT group decreased to 0.367, similar to the mortality rate of 0.345 in the EVT + SMT group, EVT + SMT would not be cost-effective. Other parameters did not result in an ICER lower than three times the per-capita GDP (Figure 3).

The scatter plot demonstrated that EVT plus SMT was cost-effective with over 99% probability under the WTP threshold of Intl.$ 57,978/QALY (Supplementary Figure 1). The acceptability curve indicated that, when the WTP threshold was approximately Intl.$ 25,700/QALY, EVT plus SMT and SMT alone had similar acceptability. EVT plus SMT became more acceptable than SMT alone when the WTP threshold exceeded Intl.$ 25,700/QALY (Supplementary Figure 2).

## 4. Discussion

Currently, three RCTs comparing EVT to SMT in patients with BAO have affirmed EVT’s effectiveness and safety in China (8, 10, 11, 26). However, no study has investigated the cost-effectiveness of EVT plus SMT vs. SMT alone in Chinese patients with BAO. Given the background of the COVID-19 pandemic and strained healthcare funding in various countries, balancing costs and possible benefits is a problem that needs to be solved. This study is the first to investigate EVT’s cost-effectiveness for patients with BAO in China and reveals that EVT is cost-effective in the country.

In 2019, the Chinese government initiated a policy for national centralized drug procurement aimed at enhancing healthcare quality. Only drugs and medical devices listed in the centralized procurement are accessible to China’s public hospitals, which provide over 80% of medical services. Drugs and medical devices should be included in the list; only cost-effective ones can be included. In China and other regions, EVT has demonstrated cost-effectiveness in treating large artery occlusions in the anterior circulation (13, 16, 27, 28). Our study’s results indicate an ICER of Intl.$ 25,670/QALY for EVT plus SMT vs. SMT alone, which is lower than three times the *per capita* GDP in 2021 China. China’s government does not specify a WTP threshold; however, the China Guidelines for Pharmacoeconomic Evaluations recommends a range of 1–3 times the *per capita* GDP as the WTP threshold, equating to 19,326 to Intl.$ 57,978/QALY (23). With the ICER of Intl.$ 27,265/QALY (29), EVT for patients with BAO is deemed cost-effective.

Several measures have been implemented to improve healthcare quality for patients with stroke in China, including the GOLDEN BRIDGE and CHANCE series of studies (30–32). Recently, the Chinese government introduced a diagnosis-related group (DRG) pricing and payment policy to meet the requirements of health insurance management and to improve healthcare system performance management (33). The DRG payment standards for cerebrovascular interventional treatment in Beijing in 2021 were Intl.$ 22,209 or Intl.$ 30,028 for those with or without complications, respectively. This implies that any excess cost will not be reimbursed (34). Our study’s cost of EVT for BAO was derived from the CNSR. It was consistent with the DRG payment standard in China, indicating that the cost of EVT for BAO patients is acceptable and that BAO patients can be guaranteed by the Chinese government.

Compared with mature cardiovascular interventional treatments, cerebrovascular interventional treatments are still emerging. With the gradual development of neurointerventional radiology technology and the application of domestic devices for thrombectomy (35), the cost may further decrease in the future. This trend is also conducive to the extensive promotion of EVT for BAO in non-first-tier cities in China. Before 2018, only a few imported stent thrombectomy devices had gained approval from the National Medical Products Administration (NMPA) of China (36). However, since 2018, the NMPA has approved over 10 types of domestically produced stent retrievers that are more cost-effective than their imported counterparts. Assuming that domestic EVT equipment matches the therapeutic efficacy of imported products, the affordability of domestic options enhances EVT acceptability. Furthermore, China’s national volume-based procurement policy has substantially reduced medical product costs (37, 38). Currently, EVT equipment is transitioning toward volume-based procurement, indicating a foreseeable reduction in EVT cost (39). Notably, hospitals in China vary in equipment selection, materials, and stroke treatment protocols, resulting in cost differences. To address this variability, we conducted both one-way and probabilistic sensitivity analyses. The results of these analyses confirmed the robustness of our conclusions.

Regarding the cost of hospitalization for stroke owing to BAO, patients with an mRS of 3–5 are believed to incur higher costs than those with an mRS of 0–2. This also explains why managing patients with BAO is more costlier than those with stroke owing to anterior circulation occlusion, as patients with AIS caused by BAO may experience a higher incidence of mRS 3–5 and mortality (7, 40). The cost of EVT is approximately Intl.$ 16,784 ± 5,312 in our institutions and Intl.$ 16,694 in the national survey. We used the cost from the national survey to represent the overall cost of EVT in China within our model. The tornado diagram shows that the costs of SMT and EVT have a minimal effect on the ICER, whereas the discrepancy in mortality between the EVT and SMT groups has the most significant impact. Considering the robustness of the results obtained from our meta-analysis of the three current RCTs in China, which indicate that EVT can improve functional prognosis and reduce mortality at 90 days in patients with BAO, we can also deem our cost-effectiveness analysis results robust. In the scenario analysis, the only instance where effectiveness data from the BEST study showed that EVT + SMT was not cost-effective can be attributed to the fact that the BEST study was stopped early owing to a high cross-group rate. Notably, EVT + SMT would be considered cost-effective if the as-treated method was employed instead of the intention-to-treat method (8). In addition, when considering the ICER by time horizon, as the survival time extends, the ICER gradually decreases, mainly because the procedure is one-off, but its benefit is long-term. The acceptability curve demonstrated that the ICER in the third year after the procedure was lower than the WTP threshold, indicating that EVT is cost-effective for patients with BAO as long as they have a life expectancy of more than 3 years. The scatter plot indicated that the probability of EVT being cost-effective is over 99% under the current WTP threshold in China. Furthermore, the acceptability curve indicated that EVT could be acceptable with a WTP threshold of Intl.$ 25,700/QALY in China, much lower than the actual WTP threshold of Intl.$ 57,978/QALY in China, reinforcing the belief that EVT is an acceptable option for patients with BAO in China.

Compared to another study on the cost-effectiveness of EVT in patients with BAO (41), our study reported a slightly higher ICER, which may be attributed to several factors. First, their study used a higher utility value than ours, which enhanced the perceived effectiveness of EVT in patients with BAO. Additionally, the cost of long-term care post-stroke was much higher in the US than in China, potentially resulting in significant cost savings associated with EVT in the US compared to China. Importantly, both their study and ours drew a similar conclusion, highlighting EVT as a potentially cost-effective option for patients with BAO, regardless of the healthcare context in the US or China. These findings offer valuable insights for decision-makers in countries with similar healthcare systems.

Notably, the proportion of patients receiving general anesthesia during the ATTENTION and BAOCHE trials was approximately 56 and 65% (10, 11), respectively, compared to only 30.7% in a study investigating the safety and efficacy of mechanical thrombectomy plus rescue therapy for intracranial large artery occlusion in the Chinese population (42). Furthermore, the proportion of patients receiving rescue treatment was 40 and 55% in the ATTENTION and BAOCHE trials, respectively, but only 19.3% in a real-world study (42). The difference can be attributed to BAO being more commonly caused by atherosclerosis than anterior circulation occlusion. A higher proportion of general anesthesia and rescue treatments results in higher costs. Therefore, to address this concern, a scenario analysis was conducted by assuming twice the current EVT cost. Even in this scenario, the ICER of EVT plus SMT vs. SMT increased from Intl.$ 27,265/QALY to Intl.$ 51,709/QALY. However, this value remained below the willingness-to-pay threshold of Intl.$ 57,978/QALY, reaffirming the robustness of our conclusions.

## 5. Limitations

This study has several limitations. First, it was based on a mathematical model rather than real-world data, and real patient-level data may offer more robust evidence. Second, the cost was partly derived from studies on anterior circulation and partly from Chinese institutions. While a one-way sensitivity analysis using a wider range, the probability sensitivity analysis, and scenario analysis based on different studies all validated the robustness of our results, data from post-EVT studies of patients with BAO might provide better accuracy. Third, the study was conducted from the perspective of the Chinese healthcare system, while a societal perspective might offer more comprehensive information. Fourth, the increased use of general anesthesia and rescue treatments during EVT procedures for BAO may lead to increased costs. Nevertheless, the conclusion remained unchanged even when the scenario analysis assumed a cost twice that of current EVT procedures. However, using cost data derived from real-world scenarios can reduce potential bias. Finally, the study was conducted using Chinese domestic data; it may only be applicable in regions with healthcare services similar to China, making it inappropriate to extrapolate the findings to patients with BAO in other regions.

## 6. Conclusion

Compared with SMT, EVT has been found to improve the prognosis of stroke caused by BAO. Considering the Chinese healthcare system, adding EVT to SMT is a cost-effective option for patients with BAO compared to SMT alone.

## Data availability statement

The original contributions presented in the study are included in the article/Supplementary materials, further inquiries can be directed to the corresponding authors.

## Ethics statement

Ethical approval was not required for the study involving humans in accordance with the local legislation and institutional requirements. Written informed consent to participate in this study was not required from the participants or the participants’ legal guardians/next of kin in accordance with the national legislation and the institutional requirements.

## Author contributions

LW: Conceptualization, Data curation, Formal analysis, Funding acquisition, Methodology, Resources, Supervision, Validation, Visualization, Writing – original draft. YY: Conceptualization, Data curation, Formal analysis, Investigation, Methodology, Project administration, Resources, Software, Supervision, Validation, Visualization, Writing – original draft. LZ: Conceptualization, Data curation, Formal analysis, Investigation, Methodology, Writing – review & editing. PX: Conceptualization, Data curation, Formal analysis, Funding acquisition, Investigation, Methodology, Writing – review & editing. XG: Formal analysis, Investigation, Methodology, Software, Validation, Writing – review & editing. YX: Data curation, Formal analysis, Funding acquisition, Investigation, Methodology, Resources, Software, Writing – review & editing. JC: Conceptualization, Data curation, Formal analysis, Investigation, Methodology, Resources, Software, Writing – review & editing. MP: Conceptualization, Formal analysis, Funding acquisition, Investigation, Project administration, Supervision, Validation, Writing – review & editing. JT: Conceptualization, Funding acquisition, Investigation, Methodology, Project administration, Software, Writing – review & editing. QG: Data curation, Formal analysis, Investigation, Project administration, Software, Writing – review & editing. RS: Data curation, Formal analysis, Funding acquisition, Project administration, Software, Writing – review & editing. YLo: Conceptualization, Data curation, Formal analysis, Investigation, Methodology, Project administration, Resources, Software, Supervision, Validation, Visualization, Writing – review & editing. YLi: Conceptualization, Data curation, Formal analysis, Funding acquisition, Investigation, Project administration, Resources, Software, Supervision, Validation, Visualization, Writing – review & editing.
