# Supplementary material for: Endovascular treatment for basilar artery occlusion: a cost-effectiveness analysis based on a meta-analysis
Source: Front Neurol. 2023 Oct 20;14:1267554. doi: 10.3389/fneur.2023.1267554 (PMC10623329; doi:10.3389/fneur.2023.1267554)
Supplement: Supplementary file 1 [file Data_Sheet_1.pdf]

RCT filter for Pubmed:

(clinical[tiab] AND trial[tiab]) OR "clinical trials as topic"[mesh] OR "clinical trial"[pt] OR random*[tiab] OR "random allocation"[mesh] OR "therapeutic use"[sh]

RCT filter for Embase:

('clinical':ti,ab AND 'trial':ti,ab) OR 'clinical trial'/exp OR random* OR 'drug therapy':lnk “

Inclusion criteria of BASICS trial

1. Symptoms and signs compatible with ischemia in the basilar artery territory.
2. Basilar artery occlusion confirmed by CTA or MRA.
3. Age 18 or older (i.e., candidates must have had their 18th birthday).
4. If IVT is considered as part of BMM, IVT has to be initiated within 4.5 hours of estimated time of basilar artery occlusion.
5. Initiation of IA therapy should be feasible within 6 hours of estimated time of basilar artery occlusion

Exclusion Criteria of BASICS trial

1. Pre-existing dependency with mRankin ≥3.
2. Females of childbearing potential who are known to be pregnant and/or lactating or who have positive pregnancy tests on admission.
3. Patients who require hemodialysis or peritoneal dialysis.
4. Other serious, advanced, or terminal illness.
5. Any other condition that the investigator feels would pose a significant hazard to the patient if IA therapy is initiated.
6. Current participation in another research drug treatment protocol (patient cannot start another experimental agent until after 90 days).
7. Informed consent is not or cannot be obtained.

Inclusion criteria of BEST trial

1. Age ≥18 years.
2. Acute ischemic stroke consistent with infarction in the basilar artery territory;
3. BAO confirmed by CTA/MRA/DSA, within 8 hours of symptom onset;
4. Written informed consent from patients or their surrogates if unable to provide consent.

Exclusion Criteria of BEST trial

1. CT or MRI evidence of cerebral hemorrhage on presentation;
2. Premorbid mRS ≥ 3 points;
3. Currently in pregnant or lactating;
4. Known serious sensitivity to radiographic contrast agents and nitinol metal;
5. Current participation in another investigation drug or device study or registry;
6. Uncontrolled hypertension defined as systolic blood pressure > 185 mmHg or diastolic blood pressure > 110 mmHg that cannot be controlled except with continuous parenteral antihypertensive medication;
7. Known hereditary or acquired hemorrhagic diathesis, coagulation factor deficiency;

or oral anticoagulant therapy with INR >1.7 or institutionally equivalent prothrombin time;
8. Baseline lab values: glucose < 50 mg/dl or > 400 mg/dl, platelets <100×109/L, or Hct<25%;
9. Arterial tortuosity that would prevent the device from reaching the target vessel;
10. Life expectancy less than 1 year;
11. History of major hemorrhage in the past 6 months;
12. Imaging evidence of significant cerebellar mass effect or acute hydrocephalus;
13. Imaging evidence of bilateral extended brainstem ischemia.

Inclusion criteria of ATTENTION trial

1. Symptoms and signs compatible with ischemia in the basilar artery territory
2. Basilar artery occlusion confirmed by CTA/MRA/DSA;
3. Age of 18 years or older;
4. Time from stroke onset to randomization within 12 hours of estimated time of basilar artery occlusion (defined as the sudden onset of stroke symptoms consistent with acute occlusion of the basilar artery (e.g. not considering any previous minor prodromal symptoms) as adjudicated by two local experienced neurologists). If symptoms of sudden deterioration are not witnessed (e.g. wake-up or unwitnessed strokes) the time that the patient was last known to be without any major neurological deficits (such as moderate or severe weakness, stupor, coma) will be used as the time of stroke onset.
5. Written informed consent;
6. National Institutes of Health Stroke Scale (NIHSS) score ≥10 at the time of neuroimaging Exclusion criteria.

Exclusion Criteria of ATTENTION trial

1. Pre-existing dependency with mRS ≥3 for patients<80 years; premorbid mRS≥1 for patients≥80 years;
2. bilateral mydriasis;
3. Pregnancy; if a woman is of childbearing potential a urine or serum beta HCG test is positive;
4. Severe contrast allergy or absolute contraindication to iodinated contrast;
5. Participation in other clinical trials;
6. Systolic pressure >185 mmHg or diastolic pressure >110 mmHg, and cannot be controlled by antihypertensive drugs;
7. Known genetic or acquired bleeding constitution, lack of anticoagulant factors, or oral anticoagulant drugs and INR > 1.7;
8. Blood glucose < 2.7 or >22.2 mmol / L; platelet count < 50×109 / L, or hematocrit < 25%;
9. Life expectancy < 1 year;
10. Patients that cannot complete 90-day follow-up (e.g. no fixed residence, overseas patients, etc.);
11. Acute ischemic cerebral infarction within 48 hours after major surgery (patients can be enrolled if more than 48 hours);

12. Premorbid cerebrovascular inflammation;
13. Premorbid nervous system disease or mental disorders hindering the assessment of the disease;
14. Imaging exclusion criteria
1) CT/MR shows intracranial hemorrhage (patients with microbleeds on MR can be included if lesion diameter ≤5mm);
2) CTA/MRA/DSA shows the artery is seriously tortuous, variability or dissection, and thrombectomy device cannot reach the target vessel;
3) PC-ASPECTS on CT/CTA-Source Images/MRI-DWI <6 for patients<80 years (<8 for patients ≥80 years);
4) CT or MR shows the cerebellar infarction with obvious space occupying effect and obvious compression of the fourth ventricle;
5) Complete bilateral thalami or bilateral brainstem infarction confirmed by CT/MR;
6) Occlusion of both anterior and posterior circulation confirmed by CTA/MRA/DSA;
7) Intracranial tumors (except small meningiomas).

Inclusion criteria of BAOCHE trial

1. Posterior circulation AIS. Patients can be randomized within 6-24 hours from symptom onset/last seen well. Patients are ineligible for intravenous thrombolytic treatment, or have received intravenous thrombolytic therapy without recanalization.
2. Occlusion (Thrombolysis in Myocardial Infarction, TIMI 0-1) of the basilar artery or intracranial segments of both vertebral arteries as evidenced by computed tomography (CT) angiography, magnetic resonance (MR) angiography or conventional angiography.
3. Age ≥ 18 and ≤ 80 years.
4. Baseline National Institutes of Health Stroke Scale (NIHSS) score obtained prior to randomization must be equal to or higher than 6 points (The initial NIHSS cut off for inclusion was ≥ 10 but was changed to ≥ 6 after 84 enrolled patients to increase recruitment rates).
5. No significant pre-stroke functional disability (modified Rankin Scale, mRS ≤ 1).
6. Patient treatable within 24 hours from symptoms onset which is considered to be the TLSW. Isolated vertigo with nausea and/or vomiting is not considered onset of symptoms.
7. Informed consent obtained from patient or acceptable patient surrogate.

Exclusion Criteria of BAOCHE trial

General exclusion criteria

1. Known hemorrhagic diathesis, coagulation factor deficiency, or oral anticoagulant therapy with international normalized ratio > 3.0.
2. Baseline platelet count < 50000/μL.
3. Baseline blood glucose < 50mg/dL or > 400mg/dL.
4. Severe, sustained hypertension (systolic blood pressure > 220 mm Hg or diastolic blood pressure > 110 mm Hg).
5. Patients receiving sedation and/or intubated patients cannot be included if baseline NIHSS is not obtained by a neurologist or emergency physician prior to sedation or

intubation.
6. Seizures at stroke onset which would preclude obtaining a baseline NIHSS.
7. Serious, advanced, or terminal illness with anticipated life expectancy of less than one year.
8. History of life threatening allergy (more than rash) to contrast medium.
9. Subjects who has received intravenous tissue plasminogen activator (t-PA) treatment beyond 4.5 hours from the beginning of the symptoms.
10. Patients with acute stroke within the first 48 hours after percutaneous cardiac, cerebrovascular interventions, and major surgery.
11. Renal insufficiency with creatinine ≥ 3 mg/dl.
12. Woman of childbearing potential who is known to be pregnant or lactating or who has a positive pregnancy test on admission.
13. Subject participating in a study involving an investigational drug or device that would impact this study.
14. Known diagnosis or clinical suspicion of cerebral vasculitis.
15. Patients with a pre-existing neurological or psychiatric disease that would confound the neurological or functional evaluations; mRS score at baseline must be ≤ 1.
16. Unlikely to be available for 90 days follow-up.

Neuroimaging exclusion criteria

1. Hypodensity amounting to a posterior circulation Acute Stroke Prognosis Early CT score (pc-ASPECTS) < 6 and Pons-midbrain-index of ≥ 3 on CT angiography source images or MR with diffusion-weighted imaging or non-contrast CT.
2. CT or MR evidence of hemorrhage (the presence of microbleeds on MRI is allowed).
3. Complete cerebellar infarct on CT or MRI with significant mass effect and compression of the fourth ventricle.
4. Complete unilateral or bilateral thalamic infarction on CT or MRI. 5. Evidence of vertebral occlusion, high grade stenosis or arterial dissection in the extracranial or intracranial segment that cannot be treated or will prevent access to the intracranial clot or excessive tortuosity of cervical vessels precluding device delivery/deployment.
6. Subjects with occlusions in both anterior and posterior circulation.
7. Evidence of intracranial tumor (except small meningioma).

Supplemental Table 1. Proportions of mRS states at month 3 in the meta-analysis

**Chinese BAO patients**		
**mRS classifications**	EVT group	SMT group
**mRS 0-2**	0.347(0.301-0.394)	0.158(0.076-0.241)
**mRS 3-5**	0.306(0.261-0.351	0.372(0.317-0.428)
**mRS 6**	0.345(0.298-0.391)	0.464(0.367-0.56)

**All BAO patients**		
**mRS classifications**	EVT group	SMT group
**mRS 0-2**	0.348(0.309-0.388)	0.199(0.100-0.297)
**mRS 3-5**	0.294(0.256-0.332)	0.341(0.270-0.411)
**mRS 6**	0.355(0.315-0.395)	0.456(0.388-0.524)

BAO, basilar artery occlusion; mRS, modified Rankin Scale; EVT, endovascular treatment. SMT, standard medical therapy.

Supplemental Table 2. Input parameters of base case analysis and ranges of one-way sensitivity analysis

Input parameters	Base case value	Range	Source
Transition probabilities			
From non-disabling to disabling[1]			
Month 4-12	0.044	/	[12]
Beyond month 12	0	/	[12]
From non-disabling to death			
Month 4-12	0.03	/	[12]
Month 13-24	0.065	/	[12]
Month 25-36	0.028	/	[12]
Month 37-48	0.051	/	[12]
Beyond month 48	0.099	/	[12]
From disabling to non-disabling			
Month 4-12	0.082	/	[12]
Beyond month 12	0	/	[12]
From disabling to death			
Month 4-12	0.115	/	[12]
Month 13-24	0.106	/	[12]
Month 25-36	0.106	/	[12]
Month 37-48	0.13	/	[12]
Beyond month 48	0.13	/	[12]
Recurrence rate of stroke			
mRS 0-2	0.1026	0.0961-0.1093	[16]
mRS 3-5	0.1418	0.1303-0.1534	[16]
Proportion of patients in recurrent stroke			
mRS 0-2	0.534	0.470-0.596	[17]
mRS 3-5	0.324	0.267-0.386	[17]
mRS 6	0.142	0.102-0.192	[17]
RR of non-stroke death for mRS 3–5	1.68	1.49–1.92	[16]

Cost (international dollars) [2]			
Additional cost of EVT	16,694	14,359- 19,299	[22]
Additional cost of IVT	3068	2897- 3247	[22]
Hospitalization for mRS 0-2	2718	2617- 2877	[23]
Hospitalization for mRS 3-5	3375	3251- 3591	[23]
Hospitalization for mRS 6	2969	2851- 3153	[23]
Annual costs of mRS 0–2	2211	2142- 2281	[22]
Annual costs of mRS 3–5	3398	3212- 3592	[22]
Cost of recurrent stroke	3768	3686- 3852	[22]
Discount rate	0.05	0-0.08	[21]
Utility			
mRS 0–2	0.76	0.69-0.82	[17]
mRS 3–5	0.21	0.17-0.26	[17]
mRS 6	0	0	[17]
Recurrent stroke	0.2	0.16-0.26	[17]

EVT, endovascular treatment. IVT, intravenous thrombolysis. mRS, modified Rankin Scale.

1. non-disabling means mRS 0–2, disabling means mRS 3–5; 2. The related cost of EVT was 68,436 Chinese Yuan (CNY) in 2019, and it would be inflated to be 69,947 CNY in 2021, taking China's healthcare consumer price index into consideration. The cost denominated in CNY would be converted to cost denominated in international dollars, at an exchange rate of 4.19, according to the purchasing power parity published by World bank. The cost of other items was calculated using the same method.

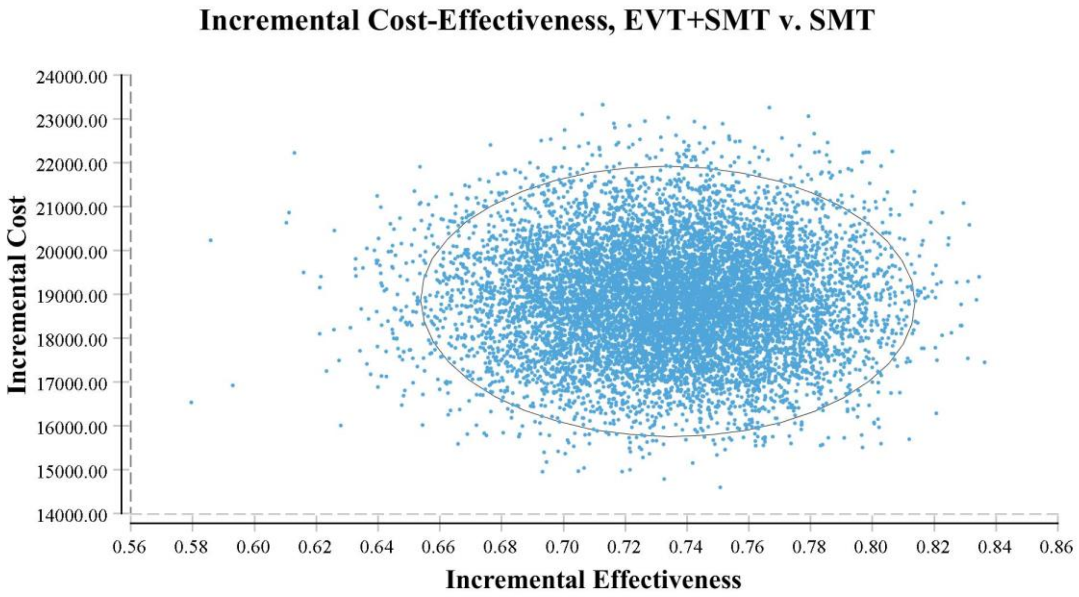

Supplemental Figure 1. Scatter plot. Endovascular treatment plus standard medical therapy was cost-effective in over 99% probability compared with standard medical therapy alone.

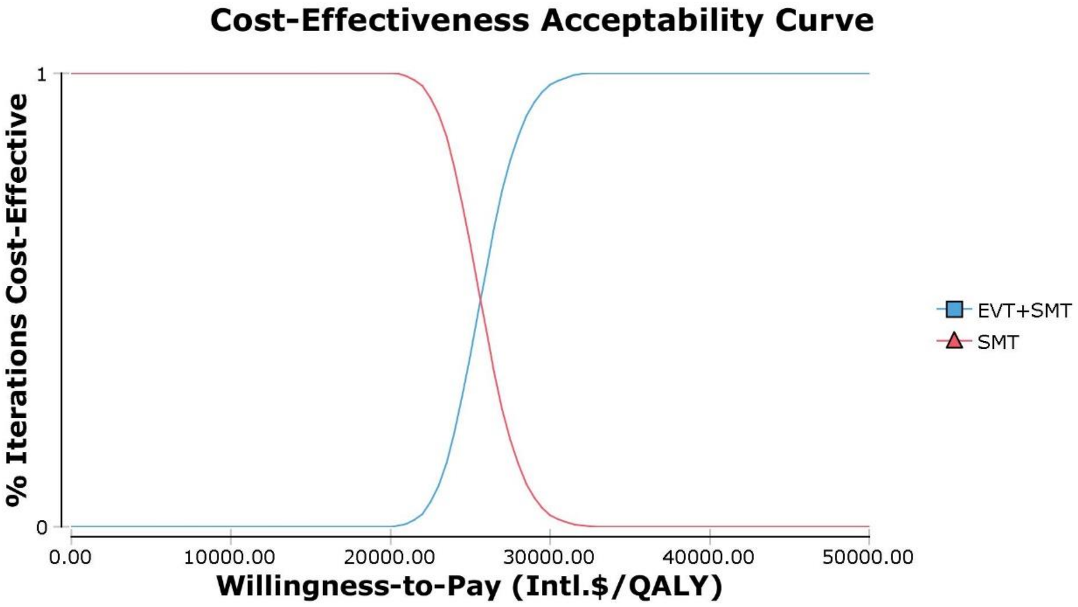

Supplemental Figure 2. Acceptability curve. When the WTP threshold was approximately 25,500 Intl.$/QALY, endovascular treatment plus standard medical therapy could gain similar acceptability with standard medical therapy, and endovascular treatment plus standard medical therapy would be more acceptable than standard medical therapy alone at a willingness-to-pay threshold of 57,978 Intl.$/QALY. WTP: willingness-to-pay; QALY: quality-adjusted life years.
